# Editorial: Expert opinions in integrative bioinformatics: 2022

**DOI:** 10.3389/fbinf.2023.1218466

**Published:** 2023-05-19

**Authors:** Tao Zeng, Chuanchao Zhang

**Affiliations:** ^1^ Guangzhou Laboratory, Guangzhou, China; ^2^ Hangzhou Institute for Advanced Study, University of Chinese Academy of Sciences, Hangzhou, China

**Keywords:** integrative bioinformatics, mendelian randomization, multi-omics, human health and disease, spatial-temporal pattern

In the era of big biological and biomedical data, the high-throughput omics data, in addition to traditional biomedical imaging data, necessitates the new integrative bioinformatics approaches to address the key challenges for the systematic understanding of human health and disease (Yu and Zeng, 2018). Along with the merging of biotechnology and information technology in precision healthcare research (Zeng et al., 2021), the integrative bioinformatics model and method opens up new possibilities for fundamental and translational research. For example, the multi-omics data and analysis have demonstrated their power in both bulk and single-cell samples (Argelaguet et al., 2019); revealed spatial-temporal functional pattern during disease early development and progression (Zhou et al., 2019); and indicated a shift in disease states between different severities of COVID-19 (Su et al., 2020). To critically evaluate the state of research in the Research Topic of integrative bioinformatics, leading specialists were invited to contribute their thoughts on latest developments and challenges, recent discoveries and future prospects in the field.

The systematic incorporation of large-scale multi-omics data is a fundamental biomedical question. Mendelian randomization (MR) provides a biomedically relevant type of causal framework to integrate big biological information for new biological discoveries. Yazdani et al. have reviewed the current advancement of the causal network models established in the theories of MR; and compared it with the classical MR framework, especially considering the transition of the MR framework to causal networks, the recognition of causal networks, the examination of the underlying assumptions as well as sensitivity and stability analysis of causal networks.

The omics-related high-throughput technologies and bioinformatic resources have great potential applications in crop breeding, which should help elucidate the interplay between gene and phenotype formation. Zhang et al. focused on addressing the ongoing status and future prospects towards integrated multi-omics approaches for predicting the complex traits of crops and deriving the regulatory networks for genetic improvement, that support intelligent crop breeding and provide cutting-edge breeding schemes for crop improvement.

Extracellular vesicles (EVs) can be secreted by almost all cell types, so most EVs are natural carriers of functional cargo and are relevant for personalized targeted therapies. To better understand the biological and biomedical properties of EVs, bioinformatic models and methods based on multi-omics data become a new frontier of integrative bioinformatic studies. Liu et al. reviewed current research on EVs and their applications using multi-omics data, and offered perspective that the integrative bioinformatic approach can reveal the temporal-spatial patterns of EVs and associated biomarkers to support a rapid response in clinical applications.

As an integrative bioinformatics study to screen candidate biomarkers and signaling pathways involved in Hepatitis B virus (HBV) associated hepatocellular carcinoma (HCC), Tan et al. used a range of analyzes on differentially expressed genes, functional pathway enrichments, protein-protein interactions, genetic alterations, prognostic factors, immune infiltrations, co-expressed genes, and miRNA-gene networks, which together suggested that ASPM upregulated in both HBV and HCC is a potential predictive biomarker for personalized treatment of HBV-related HCC.

Finally, we would like to thank all reviewers, whose great efforts ensure the high quality of all articles in this Research Topic. This Research Topic should draw more attention to the important roles and developments of integrative bioinformatics.
